# Sustainable Rabbit Production for the Caribbean: The Role of Multipurpose Trees and Forages as an Alternative Feedstuff

**DOI:** 10.3390/ani16060948

**Published:** 2026-03-18

**Authors:** Tricia Stacey Jones, Kegan Romelle Jones

**Affiliations:** 1 Department of Food Production, Faculty of Food and Agriculture, The University of the West Indies, St. Augustine Campus, Eastern Main Road, St. Augustine 999183, Trinidad and Tobago; 2 Department of Basic Veterinary Sciences, School of Veterinary Medicine, Faculty of Medical Sciences, The University of the West Indies, St. Augustine Campus, Eastern Main Road, St. Augustine 999183, Trinidad and Tobago

**Keywords:** rabbits, tropical forages, anti-nutrients, performance, digestibility, blood biochemistry, oxidative status, meat chemistry, carcass traits

## Abstract

The high demand for conventional ingredients used in the formulation of rabbit diets has increased competition, resulting in higher costs. Tropical forage plants with high nutritional content can provide relief for this pressing issue, as well as offer potential health benefits to the animal, which would ultimately lead to a quality product. A review of 33 studies on *Trichanthera gigantea* (*T. gigantea*), *Morus alba* (*M. alba*, MA), *Leucaena leucocephala* (*L. leucocephala*, LL), *Gliricidia sepium* (*G. sepium*) and *Moringa oleifera* (*M. oleifera*) and their effect on rabbits spanning 1999 to 2026 was conducted. This review demonstrated that alternative tropical fodder trees found in the Caribbean have the potential to enhance performance, the oxidative status of sera and meat, carcass traits, blood and meat biochemistry indicators and digestibility. On the contrary, most of these plants contain anti-nutrients that exert negative effects on growing rabbits and their health. The evidence revealed in this review shows that various inclusion levels of the forages can improve rabbit meat production, thereby lowering ingredient costs and providing consumers with a quality protein option. Therefore, the incorporation of tropical forages in rabbit diets is recommended.

## Simple Summary

The rising demand and resulting competition for ingredients used historically in the preparation of rabbit feeds have dramatically elevated the costs. This review revealed that locally available tropical fodder trees have high nutritional value, which could be employed to reduce production costs. Additionally, many of these plants have natural antioxidant properties that have promising potential to combat oxidative damage, thereby preventing loss of nutritional content. For example, *Moringa oleifera* (MO) and *Gliricidia sepium* (GS) have been evaluated and shown to improve antioxidant activity in both sera and meat of rabbits. Moreover, these trees have proven hypolipidemic properties that appear to improve the blood lipid profile of rabbits. The evidence presented reveals that incorporation of these tropical fodder plants in rabbit diets at various inclusion levels substantially reduces ‘bad’ cholesterol while also promoting ‘good’ cholesterol, along with increased levels of protection against lipid oxidation, which translates into a longer shelf life. Thus, these plants can offer a natural improvement in meat quality that would benefit the consumer. Therefore, it is recommended that tropical forage trees and shrubs be utilized in rabbit feeding.

## 1. Introduction

The rabbit (*Oryctolagus cuniculus*), which belongs to the order Lagomorpha, appears to be indigenous to the Iberian Peninsula and can be found in most regions worldwide, including the Caribbean [1]. They can be described as herbivorous, non-ruminants and are also classified as concentrate selectors that preferentially consume the high-protein, low-fiber, and high-carbohydrate portions of plant material [2,3].

In commercial production, rabbits will readily accept a pelleted feed [4]; however, they have thrived on diets high in roughage, with little to no grain [1]. Alfalfa meal and wheat middlings are prime components of their diet in countries like China, but in developing countries, forages such as *L. leucocephala* and *G. sepium* have greater prominence [3]. This caecum-containing mammal also directly consumes cecotrophes, expelled after cecal fermentations.

According to the FAO [5], the world population will be 9 billion by 2050, and food output will need to double by then. Rabbit production could play a vital role in sustaining the region’s food security. Currently, the demand for meat in developing countries is trending upwards, with a proportional increase in feed requirements [6]. The major operational cost in rabbit farming was determined to be feed, accounting for 64% of the intermediate consumption costs [7], which is in agreement with the feed percentages put forth by De Blas and Wiseman [8]. Intensive rabbit production requires a complete pelleted feed made of dried and ground raw materials to meet their nutrient needs. Traditionally, these conventional ingredients include the highly nutritious alfalfa, soybean, and wheat middlings. However, factors such as static production and lower availability of conventional ingredients due to the high demand from producers have increased feed prices [9]. Moreover, the feed–food competition between animals and humans [6] and high shipping costs have compounded the issue, resulting in dramatic increases in the price of imported feed and feed ingredients [10].

There is now a pressing need to reduce this cost in rabbit production. One approach for doing this is to incorporate lower-cost, nutritious, locally available high-protein forages in the feeding regime. Investigations into unconventional tropical fodder trees and shrubs are ongoing. However, the potential of these options could be diminished by anti-nutritional factors that are commonly found in plant sources [11]. This review focuses on the effects of tropical forage plants in the diet of rabbits. The effects of these feedstuffs on carcass traits, blood biochemistry, anti-oxidative activity, animal performance and digestibility will also be shown.

## 2. Methodology

This review was crafted using reports and articles sourced from scholarly publication databases (UWI linc, Google Scholar and EBSCOhost). Searches were conducted for articles in English and Spanish from the period 1999 to 2025. Initial searches yielded 110 articles. In the end, 63 studies achieved the inclusion criteria, as research on tropical trees and forages, used in rabbit meat production, under controlled experimental conditions to evaluate the different plants. Further, the studies were restricted to those utilizing a control diet for comparison against differing levels of inclusion. Data was extracted on species names (e.g., Trichanthera (*Trichanthera gigantea*), Moringa (*Moringa oleifera*), Mulberry (*Morus alba*), Gliricidia (*Gliricidia sepium*) and Leucaena (*Leucaena leucocephala*)) combined with key terms such as, rabbits, and performance using Boolean words, ‘and’ and ‘or’ to access relevant studies for this review. Qualitative synthesis was then used to compare the data.

## 3. *Trichanthera gigantea* (*T. gigantea*)

**Figure 1:**
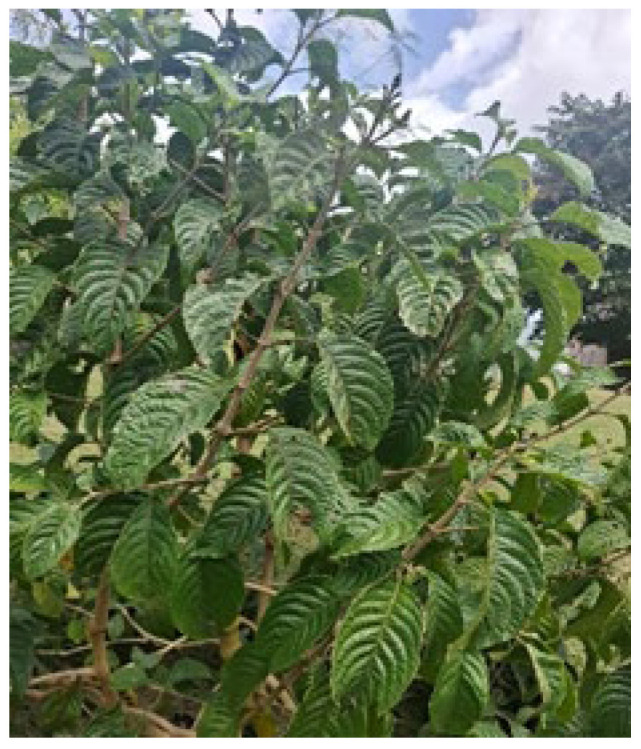
Showing *Trichanthera gigantea*.

The *T. gigantea* tree (Figure 1) is a native of South America, that generates 17 t/ha of herbage every three months [12]. It can also withstand frequent and repeated cuttings, even with a lack of fertilization [12].

### 3.1. Chemical Composition

This green fodder has a moderately high average protein content of 17.9% DM with at least 10 amino acids including Lysine (3.7%), Methionine (1.4%) and Threonine (4.3%). It is also a source of fibre with a value of 16.1% [12] which is necessary for nutrition, movement of digesta, maintaining of intestinal mucosa and controlling gut flora [7]. Calcium levels are high in comparison to other fodder trees with a value of 38.0 g/kg [12] which might be due to the presence cystoliths [12] in the leaves. Potassium content is also high at 31.8 g/kg [13] which brings to the fore its potential in feeding lactating animals [14]. Reported ADF values range from 24.5 to 56.3% of the DM. See Table 1 for chemical composition data on forages.

### 3.2. Effect on Rabbit Performance

*T. gigantea* inclusion at 15% in the diet of weaned rabbits significantly decreased (*p* < 0.05) dry matter intake by 12.94% [15]. Brenes-Soto [15] reported a significant decline (*p* < 0.05) in crude protein (CP) intake by 11.86% following increases in foliage additions to 15% inclusion. The lower CP intake observed might be linked to the lower DM intake, due to the forage being offered in fresh form. Sarwatt, Laswai, and Ubwe [16] saw a significant increase (*p* < 0.05) in daily feed intake (DFI) at 27% inclusion, which is similar in value to the outcome seen at 30% inclusion reported by [15]. Significant elevations in CP intake (49.11%) and daily weight gain (DWG) by 35.94% (*p* < 0.05) were also revealed by [16]. The higher CP intake, with a higher feed intake (FI) might likely be due to a good protein-fibre ratio in the diet, needed for digestion and growth, as such, a higher DWG was observed. Moreover, it is noteworthy that a higher FI could be linked to a lack of anti-nutritional condensed tannins [12] and low levels of alkaloids, saponins, and steroids [17]. Average daily weight gains (ADWG) reported by [15] were similar numerically to those highlighted by [16] for the 15% treatment (*p* > 0.05). This increase could be due to the protein being of good quality. The feed conversion ratio (FCR) was also reported as statistically insignificant (*p* > 0.05), compared to the conventional diet [14].

The results show that low DM content can negatively impact animal growth and health, but also the palatability of the forage can influence FI. The level of forage processing must be given attention when preparing rabbit diets, because the level of water will affect DM content. Also, the level of anti-nutrients in the forage should be assessed, since lower concentrations would not be deleterious to the animals, but higher proportions might require pre-treatment to limit poor effects.

### 3.3. Forage Substitution and Digestibility

It was observed that *T. gigantea* incorporated at 27% significantly improved crude fibre (CF) digestibility (*p* < 0.05) by 71.19% [16]. This might suggest the increased CF digestibility could be linked to a significant portion of the fibre having lower lignification [18]. Crude protein and EE digestibility showed a significant decline at *p* < 0.05 by 10.89% and 16.04% respectively [16]. The decline in EE digestibility might be due to the lower fat content in the experimental diet. The decline in CP digestibility, is possibly due to a significant level of ADF which is typical in tropical forages, and in particular *T. gigantea* which has the highest value of the forages under review, that limits nutrient digestibility [19,20]. In a trial conducted by [21] using a diet containing 30% *T. gigantea* found that the ileal digestibility of protein was not significantly different from the basal diet (*p* > 0.05). This might indicate the quality of the *T. gigantea* protein is similar to that of the control. The lower CP digestibility registered here, along with a higher DWG reported in the performance data in the same study, might be due to the increase in FI, which would have given the animal more access to the feed nutrients through higher ingestion, leading to a higher availability of nutrients for development and growth.

Also, these results show that high levels of indigestible fibre can impact CP digestibility, but CF digestibility itself is dependent on the fibre composition, in particular its level of digestible versus indigestible fractions. Therefore, attention should be given to the assessment of the fibre compositions in this forage when designing rabbit diets. In Table 2, some results are presented on maximum inclusion levels of forages and their effects on the growth and health of rabbits.

### 3.4. Carcass Traits

Carcass yields reported by [13] for the control, and the 15% treatment were not significantly different (*p* > 0.05). The protein in the treatment diet and the control might be of similar quality and utilized similarly to increase yields. Sarwatt, Laswai, and Ubwe [16] found that *T. gigantea* incorporated at 27% significantly improved (*p* < 0.05) the hot carcass weight (7.48%). The significant yields could be due to the *T. gigantea* comprising a better protein quality than the control, plus this shrub has uniquely high amounts of Ca that would support bone tissue development.

The outcome shows that the quality of the protein in the animals’ diet, inclusive of the amino acid content, as well as other essential minerals is important to improve carcass yields.

The authors suggest that a 27% *T. gigantea* could be an adequate substitution level in rabbit diets. Lower concentrations of anti-nutrients, minerals and good protein quality, together positively enhance FI, weight gain and carcass yields. Though high ADF levels documented for this forage can reduce CF digestibility and by extension CP digestibility [22]. Therefore, the quantity of insoluble fibre in this forage should be assessed along with the toxin levels, which can pose health challenges at high amounts and thus would require treatment to limit possible effects.

**Table 1:** Chemical composition of some tropical forages.

Forages	GE (MJ/kg DM)	DM (% as Fed)	CP (%DM)	EE (%DM)	CF (%DM)	NDF (%DM)	ADF (%DM)	Ash (%DM)	Ref.
*T. gigantea*	16.1	17.5	17.9	4.9	16.1	48.1	38.9	21.4	[12]
*M. oleifera*	18.6	26.2	24.3	5.4	13.6	28.3	19.3	10.3	[23]
			30.3 (dried)						[24]
*G. sepium*	19.7	25.3	22.3	4.2	19.7	49.7	34.8	10.0	[25]
*L. leucocephala*	19.0	29.9	23.3	4.0	19.9	40.9	25.4	8.5	[26]
*M. alba*	17.5	30.2	18	3.5	13.7	37	25.1	12.8	[27]
			24.05						[28]

**Table 2:** The main findings on the effect of forage feeds on rabbit performance, digestibility, oxidative status in blood and meat, biochemistry of blood and meat.

Alternative Forages	Recommended Diet %	Main Findings	Sex	Age (Days)	Breed	Ref.
*T. gigantea*	27	Significant improvements in DFI, protein intake and DWG; significant increases in CF and CP digestibility; significant decrease in EE; significant increase in HCW	M/F	42	NZW x CW	[16]
	30	Significant increases in FBW and DWG; significant decrease in DFI; no significant difference in FCR	-	-	Alex	[29]
	30	Significant increases in CP, OM and DM digestibility	-	-	Alex	[29]
	30	Significant increase in TAC; Significant decrease in MDA	-	-	Alex	[30]
*M. oleifera*	15	Significant decreases in cholesterol, total lipids and HDL; Significant decreases in SFA, cholesterol, EE; significant increases in PUFA and CP (meat)	-	28	NZM	[19]
	30	No significant differences in dressing and foreleg percentages	M	35	Alex	[31]
	1.5	Significant increase in hind legs percentage	M	35	NZW	[32]
	40	Significant increases in TWG, DWG, AFW and ADWG; significant decrease in TFI; no significant difference in FCR	-	28	California	[33]
	50	Significant increases in apparent digestibility and total digestible nutrients; significantly lower AST levels and cholesterol Significant increase in serum oxidative status	M/F	42	-	[34]
*G. sepium*	25	Significant decline in oxidative rancidity (meat)	-	42	NZW x Chin	[35]
	15	No significant differences in carcass traits	-	-	NZW	[36]
	25	No significant differences in FBW, DWG, TFI, DFI and FCR; significant increase in CF digestibility	-	-	NZW	[37]
	20	No significant differences in total protein, albumin, total cholesterol, triglycerides, LDL and HDL	M/F	56	NZW x Chin	[38]
	40	No significant differences in dressing, liver and kidney percentages	M/F	-	Dutch	[39]
*L. leucocephala*	10	Significant increases in FBW, DWG and TWG; significant decrease in FCR	M/F	56	-	[40]
	50	Significant increases in CF and ADF digestibility; no significant differences in nitrogen retention or excretion and CP digestibility	-	36–38	NZW x Chin	[41]
*M. alba*	15	Significant increase in globulins; significant increase in AST though within normal range; Significant decreases in triglycerides and cholesterol; no significant differences in TAC	-	28	Chin	[42]
	12	Significant increases in dressing percentage, HCW and CCW	-	42	Flanders	[43]

DM: Dry Matter; OM: Organic Matter; CP: Crude Protein; EE: Ether Extract (Crude Fat); ADF: Acid Detergent Fiber; CF: Crude Fibre; FI: Feed Intake; DFI: Daily Feed Intake; DWG: Daily Weight Gain; ADWG: Average Daily Weight Gain; FBW: Final Body Weight; LBW: Live Body Weight; TFI: Total Feed Intake; TWG: Total Weight Gain; FCR: Feed Conversion Ratio; HDL: High Density Lipoprotein; LDL: Low Density Lipoprotein; TAC: Total Antioxidant Capacity; FA: Fatty Acids; PUFA: polyunsaturated fatty acids; SFA: saturated fatty acids; AST: Aspartate Amino Transferase NZW: New Zealand White; CW: California White; Chin: Chincilla.

## 4. *Moringa oleifera* (*M. oleifera*)

Indigenous to the Himalayas, *M. oleifera* trees can now also be found in tropical and subtropical regions. Growth rate of this plant is exceedingly high, with first harvest of foliage starting at 2.5 months. Yields of fresh matter can vary from 27 to 120 tonnes/ha for the first cutting, with up to 9 harvests/year [23].

### 4.1. Chemical Composition

**Figure 2:**
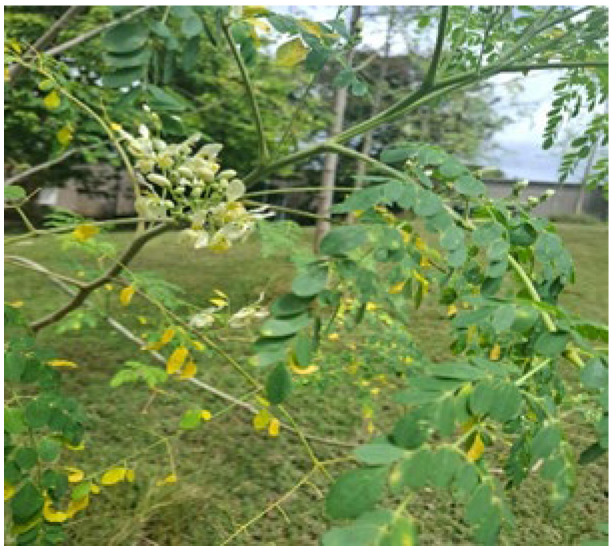
Showing *Morinaga oleifera*.

*M. oleifera* trees (Figure 2) are characterized by high CP content with values up to 30.3% DM, and an average ADF value of 19.3% DM. There are also 19 amino acids inclusive of lysine (4.8% protein), theronine (4.4% protein) and glutamic acid (11.5% protein), minerals including high average levels of Ca (26.5 g/kg DM) and Fe (497 mg/kg DM), fatty acids including significant amounts of α-Linolenic acid (44.57%) in dried leaves, vitamins such as B1, B6 and ascorbic acid, as well as, flavonoids (quercetin and kaempferol) [23] make it a good candidate as a feed source for rabbits. Lignin levels average 7.0% DM.

### 4.2. Effect on Rabbit Performance

A study saw significant increases (*p* < 0.05) in DFI (13.37%), ADWG (23.55%), and FBW (11.91%) at the 1.5% inclusion level for MO in the diet [44]. Significant improvements in growth parameters were observed for the 6% inclusion by El-Desoky et al. [45]. In particular, a significant decrease in the FCR (27.01%) and significant increases seen in TBWG (26.64%) and DWG (44.91%) over the control (*p* < 0.05). While no significant differences (*p* > 0.05) were seen in LBW and DFI for the 6% MO diet. Selim et al. [31] received significant increases (*p* < 0.05) in DWG (27.09%) and FBW (15.31%), while DFI produced no significant difference (*p* > 0.05) and FCR significantly lowered by 20.70% for the 1.5% inclusion diet (*p* < 0.05). Another trial found no significant differences (*p* > 0.05) between the control diet and the 30% MO inclusion diet for FBW, DWG and FCR, while the 40% MO diet had a significant decline (*p* < 0.05) in FBW and DWG by 5.93% and 8.86% respectively, as well as, a significant increase in FCR (11.76%) compared to the control [29]. However, the control and the 40% MO diet had similar DFI (*p* > 0.05), while the 30% MO diet was significantly lower by 12.21% compared to the 40% MO diet (*p* < 0.05).

Improved performance indices might be due to good digestibility of the CP, and a trove of nutrients embodied in *M. oleifera*. The presence of essential amino acids, minerals and other plant-based phytonutrient compounds in rabbit diets can impact performance outcomes. Though *M. oleifera* like other legumes contain anti-nutrients, including phytates that bind to essential minerals, thus reducing their bioavailability for absorption [46]. Therefore, consideration should be given to these plant compounds that could inhibit animal development.

### 4.3. Forage Substitution and Digestibility

Mohammed et al. [44] conducted studies on rabbits using MO to replace the basal diet at rates of 0.5%, 1% and 1.5% in treatments M1, M2 and M3 respectively. The report declared significantly high (*p* ≤ 0.05) digestibility for DM, OM, CF, EE, and NFE, but the CP value was insignificantly different to the control [44]. An in-depth look, showed digestibility coefficients of DM and OM for M3 surpassed all other treatments and the control with increases of 11.73 and 13.11% respectively. The M3 diet also produced the highest digestibility figures with increases of 10.11 and 9.29% for EE and NFE respectively. Hafsa et al. [19] saw a similar trend when 15% MO was substituted in the diet, showing CP digestibility significantly increased (*p* < 0.05) by over 3%, as well as 3% increases for both OM and DM.

This indicates the *M. oleifera* diet contained lower amounts of lignin, thus improving digestibility, which is supported by literature evidence showing this forage has the lowest average ADF, compared to other common tropical fodder trees reviewed. The level of fibre digestibility is dependent on the insoluble fibre content, which ultimately impacts CP digestibility and should not be disregarded in feed optimization.

### 4.4. Blood Biochemistry

The blood chemistry data show promising results for both the lipid profile and anti-oxidative activity of MO, at both low and high incorporations. The study conducted by Jimoh et al. [47] using a 5% inclusion level, reported significant decreases (*p* < 0.05) in cholesterol (3.59%), and LDL (6.06%) compared to the basal diet. But, total protein, albumin and globulin were significantly increased (*p* < 0.05) by 33.06, 42.39 and 27.50% respectively. A significant increase (*p* < 0.05) of 32.65% for the AST activity was also observed in this study, but is still within normal range indicating *M. oleifera* does not have a detrimental effect on liver health [48]. Selim [31] saw a similar trend with increased values for total protein (9.84%) and globulin (36.00%) at 1.5% inclusion (*p* < 0.01), while AST data showed a significant decline by 15.44% (*p* < 0.01). The higher serum total protein and globulin levels could be due to the presence of flavonoids that are reported to have hepatoprotective effects [49]. Moreover, greater quantities of globulin are known to enhance immunity [50].

Salem et al. [30] found that 20 and 30% inclusion levels, produced significant declines (*p* < 0.05) for cholesterol (9.82%) and total lipids (6.67%) for 20% inclusion, while the 30% level showed a drop by 11.01% (cholesterol) and 8.37% (total lipids) respectively. The 20% treatment had an additional benefit by producing a significant (*p* < 0.05) boost to HDL by 13.77% which was not significantly different to the 30% treatment (*p* > 0.05). The general downward trend in cholesterol levels suggests a hypocholesterolemic effect by the presence of saponins found in *M. oleifera* [51] that was shown to reduce cholesterol [47].

These results demonstrate that *M. oleifera* has some impact on immunity, liver health and cholesterol levels. Special focus should be given to these encouraging effects in the preparation of rabbit diets, if improvement in animal health is a desired outcome.

### 4.5. Oxidative Status

Rabbit sera data reported by [30] hinted at an improvement in antioxidant activity at both low and high inclusion levels. At levels up to 30% MO, there was a significant reduction in MDA (19.79%) and a significant increase in total antioxidant capacity (30.23%) at *p* < 0.05. The presence of flavonoids in *M. olifera* might be instrumental in reducing oxidative stress which is expressed as lower MDA concentration, while also boosting antioxidant levels [52]. The presence of flavonoids has an impact on antioxidant activity and this effect is valuable, if rabbit health is a primary focus, when formulating animal diets.

### 4.6. Meat Chemistry

Meat lipid measurements conducted by Selim [31] showed a significant drop in cholesterol (9.91%) at 1.5% MO replacement (*p* < 0.05). The study also discovered a significant reduction in saturated FA (37.95%), along with an opposite positive effect on polyunsaturated FA that was significantly increased to 32.45% (*p* < 0.05). The elevated level of PUFA in the meat, might be due to the higher PUFA content in *M. olifera* [53] which can be deposited directly into the meat through the diet [54]. Data on the meat chemical composition showed a significant (*p* < 0.05) increase in CP (6.26%) and decrease in EE (2.26%), while the ash content was similar to the control (*p* > 0.05). These findings suggest that *M. oleifera* could impact the proximate analysis of meat.

The increased PUFA content in the meat, particularly the n-3 PUFA is impacted by the PUFA content in *M. oleifera*, which contains 26.46% [53] of the essential α-Linolenic acid. This result can provide a better meat quality to customers, in terms of the potential health benefits, by the lowering of SFA and the increase in immunity and cardiovascular health linked to n-3 PUFA [55]. But, a higher PUFA content could mean greater lipid peroxidation resulting in lower shelf- life. However, the presence flavonoids like quercetin and kaempferol, have significant antioxidant effects, which is superior to ascorbic acid [56].

Greater concentrations of PUFA in *M. oleifera* can impact the PUFA quantity and quality and lower SFA. If the approach is to produce healthier meat for consumption, then dietary inclusion of *M. oleifera* should be promoted.

### 4.7. Carcass Traits

In the realm of carcass traits, investigations done by [29] into carcass characteristics revealed similar dressing weight percentages and fore leg percentages for the rabbits on both the 30 and 40% diets (*p* > 0.05). The breast and rib percentages seen at the 30% level was shown to be significantly higher (*p* < 0.05) than the 40% MO diet by 9.62%. However, the 40% level dominated over the control (*p* < 0.05) for the hind legs percentage by 37.19%, while maintaining no significant difference with the 30% treatment (*p* > 0.05). William et al. [57] reported significant increases (*p* < 0.05) in both hot carcass weight (HCW) and cold carcass weight (CCW) at the 15% level by 50.46 and 58.31% respectively. While the hind legs percentage on the 15% treatment were not significantly different to the control (*p* > 0.05).

The quercetin content in *M. olifera* could be responsible for muscle development [52] leading to improvements in several carcass parameters. Therefore, the level of flavanols in the diet could enhance muscle development leading to better carcass traits.

An assessment of the data suggests that *M. olifera* can replace the basal diet of rabbits up to 30%. Improved weight parameters linked to generous amounts of nutrients and good digestibility of CP supported by low ADF, in the forage can be reversed by anti-nutrients like phytates, at high levels leading to lower outcomes in growth parameters. On the other hand, the presence of compounds like saponins and flavonoids have shown to enhance lipid profiles, liver health and immunity, without hampering the well-being of growing rabbits. However, a cautionary approach should be adopted regarding the incorporation of high proportions of this forage, without treatment to control the anti-nutrient content, since this could have deleterious effects on rabbit health.

## 5. *Gliricidia sepium* (*G. sepium*)

*G. sepium* is a native species to Central America, but can now be located throughout the tropical region. This legume can generate 9 to 16 tonnes/ha of DM, with first harvest possible 7 months after establishment of cuttings or 14 months after seedling. Harvesting frequency is every 2 to 3 months during the wet season and every 3 to 4 months in the drier period [25].

### 5.1. Chemical Composition

**Figure 3:**
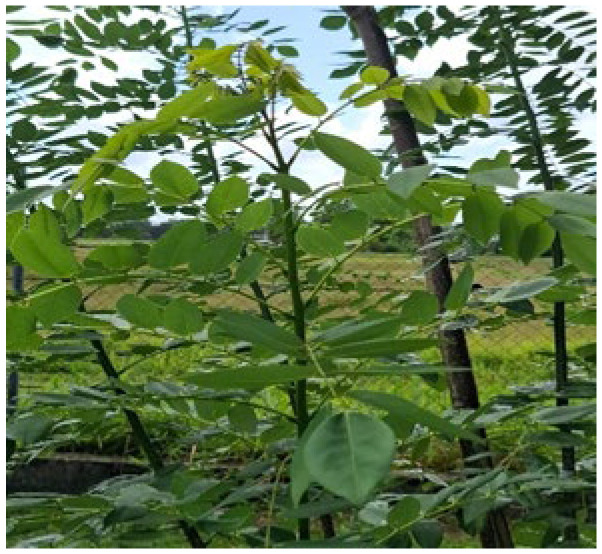
Showing *Gliricidia sepium*.

The tropical forage tree, *G. sepium* (Figure 3) has a relatively high average CP content of 22.3% DM, as well as, average DM, CF, and ADF with percentages of 25.3, 19.7, and 34.8 DM respectively [25]. Minerals such as Ca, Fe, Mn and Zn had average values of 11.9 g/kg, 153 mg/kg, 79 mg/kg and 35 mg/kg. Tannins content can reach as high as 52.8 g/kg in addition to unidentified alkaloids and hydrogen cyanide (HCN) that can peak at 4 mg/kg. Coumarin detected in this tree can increase *G. sepium*’s toxicity due to bacterial action that can produce dicoumerol upon fermentation. This forage’s lignin content was also reported to range from 4.5 to 22.2% DM [25].

### 5.2. Effect on Rabbit Performance

In a study conducted by [35] on crossbred New Zealand and Chinchilla rabbits, the inclusion of 15% GS did not have a negative impact on DWG, DFI and FCR (*p* > 0.05). In both the Odedire and Abegunde [58] experiment that used 75% inclusion and the Akin-Aina et al. [33] trials that incorporated 50% GS along with the same control (*Centrosema pubescens*) saw significant increases in several performance parameters. In the case of [58], significant increases (*p* < 0.05) were seen for TWG (15.15%) and ADWG (15.15%) and significant decreases in TFI, ADFI and FCR by 15.84, 13.67 and 14.94% respectively. Akin-Aina et al. [33] had a similar trend in results, with increases (*p* < 0.05) in TWG and DWG by 15.15 and 15.49% respectively. TFI also declined by 10.05% (*p* < 0.05), but FCR was not significantly different to the control (*p* > 0.05). Dada [32] found that a diet containing 50% GS was significantly better versus a diet containing 75% GS (*p* < 0.05) for AFW (11.36%), ADWG (30.44%) and AFI (36.30%).

The lower FI intake observed in a couple of trials could be due to the presence of condensed tannins, hydrogen cyanide (HCN), coumarin or alkaloids that reduces palatability. This outcome supports the literature that some animals cannot tolerate high levels of anti-nutritional factors very well [49]. Hence the reason the FI intake dropped, which is possibility due to a lower palatability. However, the higher weight gains over the control is possibly due to the high digestibility of the forage which releases nutrients to support the animals’ growth. Both the anti-nutritional content and digestibility of the diet can impact performance parameters. Anti-nutritional levels should be factored in when creating diets and efforts should be made to reduce their content, to minimize drawbacks in FI.

### 5.3. Forage Substitution and Digestibility

Adejumo [34] saw a significant increase (*p* < 0.05) in the apparent digestibility percentage in a cassava-herbage diet containing 25% *G. sepium* versus a blend containing 33% *G. sepium* by 11.42%. Total digestible nutrients were on trend with the 25% GS treatment having a 9.16% advantage over the 33% GS diet (*p* < 0.05). The better digestibility indicates a diet that is nutritionally better balanced.

Akin-Aina et al. [33] saw a significant decline (*p* < 0.05) in DM digestibility by 60.19% for 50% GS inclusion. *G. sepium*’s high CF content, along with a high ADF value, could also have impacted DM digestibility. Significant increases (*p* < 0.05) in fecal CP (4.88%), fecal nitrogen (4.93%) and urine nitrogen (25.00%) were also observed [33]. High fecal CP and urine nitrogen is possibly linked to *G. sepium*’s high CP content, particularly due to its nitrogen-fixing capability, that could have exceeded the metabolic capacity of the animals. But, ref. [58] saw nitrogen utilization, faecal nitrogen and urine nitrogen were all similar to the control (*p* > 0.05), indicating that the *G. sepium* protein content is metabolized well, in the rabbits, at the 75% level.

The CF composition and CP levels can impact digestibility. In the formulation of rabbit diets there is need for proper CP and CF balances, with particular attention to be paid to the quality of the fibre, which is partially dependent on the age, season and frequency of harvesting that can influence lignin, cellulose and hemicellulose proportions.

### 5.4. Blood Biochemistry

The effect of *G. sepium* on the lipid profile produced encouraging results in the study by [35]. There was a significant triple decline (*p* < 0.05) in triglycerides (5.98%), cholesterol (47.87%) and LDL (78.83%) for the 15% GS diet. The lowering of blood lipids could be attributed to bioactive compounds in this legume forage. Saponins that are present in *G. sepium* have been shown to inhibit cholesterol uptake in the gut [49]. Amata [33] revealed a significant increase (*p* < 0.05) in total proteins by 15.53% on the 20% GS diet and a significant decline (*p* < 0.05) in albumins by 3.87%. These results indicate possible issues with liver function, but there was no significant differences in globulins. Similar levels of globulins indicate the treatment diet and control contribute comparable levels of immunity. The study that used sun-dried forages showed the AST level of the cassava-herbage mix containing 25% GS was significantly lower (*p* < 0.05) by 37.18% to the control and 18.91% to the other treatment containing 33% GS [34]. This indicates a possible effect by coumarin, which is present in significant amounts in *G. sepium*, that have shown to offer protective benefits to the liver of rabbits [59]. Cholesterol levels were also better for the treatments (*p* < 0.05) versus the control by 13.02% (33% GS treatment) and 4.07% (25% GS treatment), which mirrored the outcome seen by [35]. Bioactive compounds in *G. sepium* have an impact on blood parameters, and their levels should be given attention. While their presence has some potential health benefits, a balance should be struck regarding their amounts, as high levels could reduce any positive effects.

### 5.5. Oxidative Status

Significant increases (*p* < 0.05) in superoxide dismutase and GPx by 49.32 and 30.95% respectively at 15% inclusion of GS were reported by [35]. An improvement in antioxidant activity could be due to antioxidant properties of the forage, possibly saponins which have been shown to have antioxidant activity [49].

Saponins have an impact on oxidation status and their inclusion in rabbit diets could improve the animals’ health, since they play a role in antioxidant defense systems, to combat free radicals, which are often linked to disease.

### 5.6. Meat Chemistry

At 25% GS inclusion Oshibanjo et al. [36] saw an improvement of meat quality, via a significant decline (*p* < 0.05) in oxidative rancidity by 43.88%. Saponins in *G. sepium* might be responsible for a reduction in lipid oxidation, due to its antioxidant activity [51]. Saponins have an impact on the oxidative status of meat, but appropriate levels need to be present to reduce meat degradation and extend shelf life.

### 5.7. Carcass Traits

Amata [37] used GS up to 20% in the diet and found no significant differences in all of the carcass traits, inclusive of dressing percentage (*p* > 0.05). This outcome is possibly due to a closely matched nutrient content profile with the control, which metabolized in similar fashion. The quality of protein and other minerals like Ca and Zn, present would have resulted in comparable muscle and bone development and are important considerations for carcass gains [60].

The authors suggest a 50% inclusion rate for *G. sepium* as a partial substitute in rabbit diets. The improved weight gains indicate a forage that is highly digestible, but these increases are tempered by the effects of anti-nutrient levels like, HCN and coumarin that can reduce palatability, thus hindering FI. While metabolites associated with this plant can act as natural boosters, improving several serum indicators, their levels must be addressed with pre-treatment to ensure their positive effect is not diminished by excessively high levels.

## 6. *Leucaena leucocephala* (*L. leucocephala*)

*L. leucocephala* is indigenous to Guatemala and Mexico and can also be found across the Asian Pacific region, including Australia and the Caribbean. Annual yields can reach a maximum of 30 tonnes/ha of DM. Harvest intervals can be as short as 6 to 8 weeks or longer at 12 weeks, which is dependent on the productivity of the site [26].

### 6.1. Chemical Composition

**Figure 4:**
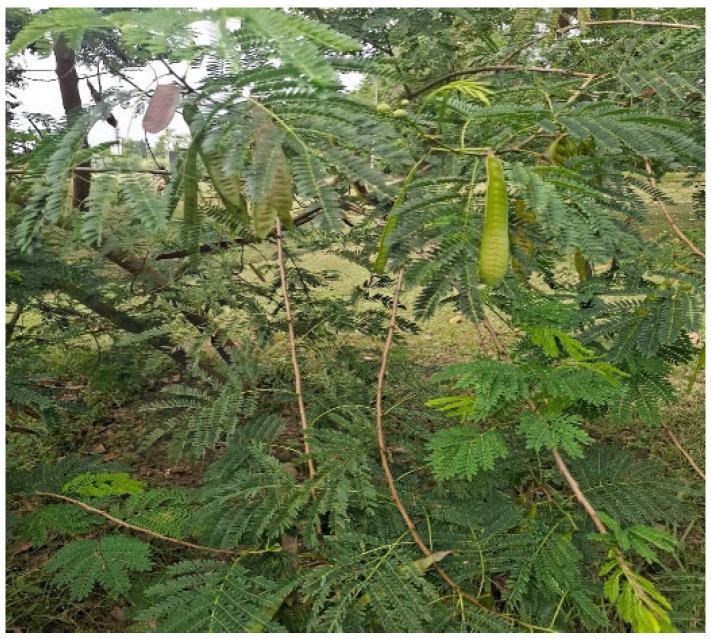
Showing *Leucaena leucocephala*.

*L. leucocephala* is a leguminous shrub (Figure 4) with a CP content averaging 23.3% DM. In addition, average DM, CF, and ADF were recorded at 29.9, 19.9 and 25.4% DM. Minerals such as Fe, Mn and Zn had average values of 261, 65 and 30 mg/kg. Ligin in the shrub ranged from 4.5 to 22.0% DM. Notwithstanding the high protein content, high levels of the toxic, non-protein amino acid, mimosine, found in large concentrations in *L. Leucocephala*, must be considered. Especially, when recommending dry matter intake of this forage for non-ruminants, that are more likely to suffer negative effects [26].

### 6.2. Effect on Rabbit Performance

Growth performance factors such as FBW and FCR got a significant boost (*p* < 0.05) at both 10% and 30% inclusion of fresh *L. leucocephala* stems and leaves, in trials done by [40] and [61] by 8.69 and 15.41% respectively and significant declines in FCR by 21.20 and 35.95% respectively (*p* < 0.05). This result is possibly due to a quality CP content that is highly digestible. R. Adedokun et al. [62] also saw significant increases (*p* < 0.05) in ADFI (24.25%) and ADWG (59.06%) on the 10% LL diet. The heightened DFI and resulting weight gain might be due to oven-drying at 30 °C until dryness which reduced mimosine and increased palatability. Wiratmini et al. [61] also reported a similar significant increase (*p* < 0.05) in DFI by 9.24% at 30% inclusion. Adekojo et al. [38] used a 40% replacement with pretreated *L. leucocephala* and found FBW, DWG, TFI, DFI and FCR were unaffected (*p* > 0.05) by variations in the level of dietary LL. This outcome might be due to air-drying used as a pre-treatment, that minimized the effects of the anti-nutritional factors such as mimosine [26].

The nutrient content and the anti-nutritional content can impact specific growth parameters. The improvement of rabbit weight and FI intake is dependent on the CP quantity and quality and also the level of mimosine which can hinder growth. Pre-treatment involving chemical agents such as FeCl_3_, drying and soaking in water might be necessary to increase the benefits from this forage [26].

### 6.3. Forage Substitution and Digestibility

Debnath et al. [63] attained a significant increase (*p* < 0.05) in CF digestibility by 28.35% at 10% inclusion, which was in agreement with the results from the Adekojo et al. [38] study that saw an increase by 19.76% when 40% LL in the diet was used (*p* < 0.05). But this was in opposition with the trial by [64], which saw a decrease in CF (31.4%) at 25% inclusion, among other parameters that trended in the same direction was a decline in ADF by 19.69% (*p* < 0.05). This could be related to the presence of mimosine, that has a negative effect on digestibility of dry matter and protein, especially in un-treated forage [26].

Mimosine has an impact on CF digestibility, which must be addressed. Pre-treatment is necessary to lower its content, thereby minimizing any deleterious effects on growing rabbits [26].

### 6.4. Blood Biochemistry

No significant difference in cholesterol was reported by Rohilla et al. [65] for all treatments- 0, 20, 40 and 60%, though decreasing values were observed at higher replacements of *L. leucocephala* (*p* > 0.05). Makinde [40] using a 10% LL diet saw no significant differences (*p* > 0.05) in total protein or albumin, while [65] saw a significant drop (*p* < 0.05) in albumin levels for all treatments versus the control with the 60% treatment having the lowest decline at 37.04%. This significant drop in albumin could be indicative of liver damage due to the toxicity of mimosine. Another study which replaced the basal diet with *L. leucocephala* in two treatments consisting of 25% (LL25) and 50% (LL50), saw no significant differences (*p* > 0.05) in several serum parameters, though there were numerical increases for total protein and albumin and a decline in total cholesterol for LL25, in comparison to the control [39]. Triglycerides, LDL and HDL were also similar (*p* > 0.05) but dropped to the lowest level for LL50. Contrarily, LL25 had the highest HDL (*p* > 0.05). This drop in triglycerides and LDL and increase in HDL at LL25 could be due to hypolipidemic effect of mimosine [66]. No significant differences for total protein in the studies by [39,40] indicate that the diets might be of good quality, and has no deleterious effects on liver function.

Mimosine levels have an impact on lipids and liver function. To derive the benefits from mimosine the quantities must be managed, as high levels can cause liver damage when fed to rabbits [67].

### 6.5. Carcass Traits

The percentages for dressed carcass, liver, and kidney put forward by [40] for 10% LL inclusion were not influenced (*p* > 0.05) by the dietary treatments. However, a significant decline (*p* < 0.05) was seen in dressed percentage by 8.87% when *L. leucocephala* was increased to 20% inclusion, as well as significant increases in liver and kidney by 52.13 and 42.62% respectively (*p* < 0.05). This might be due to mimosine which can decrease yields [26]. Also, elevated organ weights are a sign of increased metabolic rates in an attempt to eliminate toxic compounds, like mimosine and tannins that are inherent in *L. leucocephala*. Anti-nutrients can impact carcass yields, and their levels should be reduced via pre-treatment, if the goal is to improve carcass traits.

Overall observations suggest that *L. leucocephala* can be strategically incorporated as a partial substitute in rabbit diets up to 40%. However, the reduction of mimosine levels via pre-treatment methods is paramount, to circumvent reductions in growth and digestibility indicators. Further, concentrations of plant toxins in this forage should be managed, to derive the best improvement in serum parameters and carcass traits, without jeopardizing animal health.

## 7. *Morus alba* (*M. alba*)

*M. alba* is native to China, but is now widespread in tropical and temperate regions, and even the sub-arctic. Yields of fresh foliage range from 6.5–33.5 tonnes/ha [27]. Recommended harvest intervals are set at 2–6 months, with the best nutritive value at short cutting intervals [68].

### 7.1. Chemical Composition

**Figure 5:**
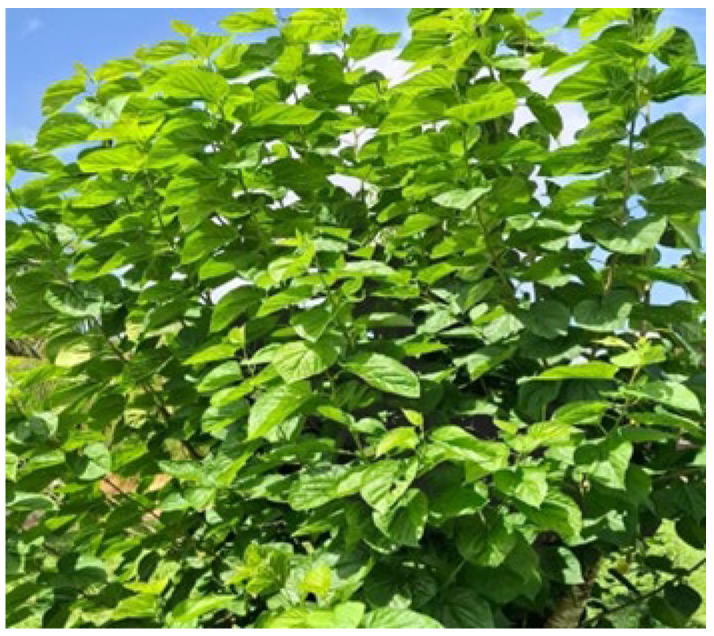
Showing *Morus alba*.

*M. alba* is a perennial tree (Figure 5) that has a relatively high average protein content of 19.4% and percentages of DM, CF and ADF at 90.5, 13.7 and 25.1 respectively [27]. Simbaya et al. [28] reported an even higher CP value of 24.05%. It also contains a high mineral content which can exceed 20% DM dominating the macro-mineral content of other trees, namely Ca (1.4–3.6% DM) and vitamins with appreciable amounts of B and C (0.3% DM). Anti-nutritional compounds, including condensed tannins are present in this plant, achieving a maximum level of 20 g/kg DM [27].

### 7.2. Effect on Rabbit Performance

Separate feeding studies by [41,69] using *M. alba* as a partial replacement for the conventional feed, using rates of 20 (MA20) and 50% (MA50) respectively, produced significant increases (*p* < 0.05) in a couple of performance parameters- FBW (4.56 and 3.51% respectively) and DWG (9.38 and 5.03% respectively). Superior weight gains over the control is possibly due to elevated amounts of minerals, vitamins and moderate protein content that will support growth. Khan et al. [41] recorded a significantly lower (*p* < 0.05) FCR (5.86%) but [69] saw no significant difference (*p* > 0.05). Simbaya et al. [28], like [41] used MA50, with both having some success in TWG with significant increases (*p* < 0.05)- 5.12% [65] and 97.98% [28]. However, Simbaya et al. [28] revealed a significant decline (*p* < 0.05) in TFI by 15.97% and a significant reduction (*p* < 0.05) in FCR (57.60%). The low intake seen with high input amounts of the forage, could be due to anti-nutritional compounds such as tannins, oxalic acid and alkaloids that have been detected in this plant [27,51]. Hou et al. [70] reported no significant increases (*p* > 0.05) in ADG, ADFI and FCR at 15% inclusion in the diet. Khan et al. [41] reported similar (*p* > 0.05) DM intake among treatments (0%, 25%, 50%, 75%), except for the 100% MA diet, which had the significantly lowest (*p* < 0.05) consumption (4.44%). This outcome also reflected the results achieved by López et al. [71] whereby a lower performance was linked to a 100% *M. alba* diet.

Anti-nutrients have an impact on performance indicators, and their levels must be adjusted through pre-treatment to avoid compromising of the FI intake.

### 7.3. Forage Substitution and Digestibility

Hassanien et al. [43] reported significant declines (*p* < 0.05) in DM (5.37%) and OM (5.56%) digestibility at 12% inclusion of MA compared to the control. This could be due to a higher percentage of lignin due to maturity of the forage. On the contrary, digestibility results by [38] showed significant increases (*p* < 0.05) for CF (71.40%) and ADF (98.21%) for the 15% MA inclusion. The improved CF and ADF digestion was possibly due to a significant proportion of the fibre being present in unlignified form. EE percentage in the study by [69] went in a positive trajectory, significantly increasing (*p* < 0.05) by 6.33% at 20% MA inclusion which was in agreement with [43] at 12% MA inclusion (19.36%) at *p* < 0.05). The higher EE digestibility could be based on the higher dietary EE content in the 15% MA diet versus the control. The studies by [42,69] did not show any significant differences in either nitrogen retention or excretion (*p* > 0.05). The similar nitrogen retentions might be indicative of comparable production of microbial protein. Prasad et al. [42] had a similar outcome to [43,69] with CP digestibility showing no significant differences (*p* > 0.05). This is possibly due to the quality of the protein being similar to the respective controls. Thi et al. [72] found no significant differences in ADF and NDF digestibility when up to 50% MA was used to replace the control diet (*p* > 0.05). Other digestibility parameters in the [72] study saw significant declines *p* < 0.05) in DM (14.42%), OM (14.34%), and CP (31.52%) versus the control. This might be due to the presence of condensed tannins [27] that are known to hinder cellulose digestion and protein digestibility [49]. The presence of anti-nutrients in the diet have an impact on digestibility indicators. The proportion of anti-nutrients in the diet should be under consideration, if fibre and protein digestibility are of significant importance in rabbit feeding.

### 7.4. Blood Biochemistry

Hassanien et al. [43] found a significant increase *p* < 0.05) in the globulin value (24.13%) at 12% MA inclusion compared to the control. Both values are above normal range [48] which might be due to the presence of tannins that can increase the formation of immunoglobulin proteins [73]. The AST value was significantly higher *p* < 0.05) by 11.83% though it remained within the normal range [48,74]. Cholesterol, and triglycerides both dropped significantly *p* < 0.05) by 15.89% and 18.56% respectively. This reduction might be due to secondary metabolites like tannins which are known to have hypocholestrolemic effects [75]. Total proteins and albumin measurements were all within normal range [48,74] and produced no significant differences (*p* > 0.05. This shows that liver function was similar across treatments. Secondary plant metabolites have an impact on several blood parameters and their concentrations should be monitored if improvements in these blood parameters are to be realized.

### 7.5. Oxidative Status

Hassanien et al. [43] observed no significant differences (*p* > 0.05) in total antioxidant capacity (TAC) at 12% MA inclusion. This might be due to the presence of polyphenolic compounds and flavonoids, such as quercetin, with natural antioxidant effect [76,77]. Phytochemicals have an impact on antioxidant activity and can improve rabbit health.

### 7.6. Carcass Traits

Hassanien et al. [43] reported no significant differences (*p* > 0.05) in HCW and dressing percentages at the 12% MA inclusion. Hou et al. [70] also had no significant increase (*p* > 0.05) in FBW, but observed a significant decline *p* < 0.05) in abdominal fat % (35.64) at 10% MA inclusion. However, Khan et al. [41] reported positive carcass trait indicators that aligned with the significant results achieved for the growth parameters highlighted with the 50% replacement diet. HCW, CCW and dressing % were improved (*p* < 0.05) by 3.03, 4.66, and 3.14% respectively. Osman et al. [78] also saw a significant increase *p* < 0.05) in dressing percentage (6.74%) at 20% MA inclusion similar to [43]. The improved carcass traits observed might be due to a quality blend of nutrients in *M. alba* that aids muscle and bone development. Nutrient quality can impact carcass development and should be a factor to consider when developing diets to boost carcass traits.

The authors recommend 50% *M. alba* substitution in rabbit feeding. Positive growth indicators have been achieved at this level, likely due to high amounts of minerals, vitamins and a considerable protein content, but, bioactive compounds like tannins and oxalic acid in the forage could diminish FI and digestibility parameters, if left untreated.

Secondary metabolites can however improve serum and oxidative status parameters, but their concentrations need to be managed to derive maximum benefit without compromising animal health.

## 8. Evaluation of Tropical Forages

Evaluations of the reviewed alternatives showed substantial differences, in their balance between nutritional value, performance effects, digestibility outcomes, blood and meat benefits, as well as carcass results. *T. gigantea* has several advantages, including a lack of condensed tannins and low levels of other secondary metabolites, moderate protein content with functional amino acids and other minerals to support growth of a quality meat product, but the relatively high indigestible fibre content could lower protein digestibility thereby limiting inclusion levels. *G. sepium* on the other hand has some challenges with toxins such as cyanogens, tannins, coumarin and other unidentified alkaloids that could reduce palatability resulting in lower FI, despite having a relatively high CP and mineral content that would benefit growing animals. It should be highlighted that some anti-nutrients did produce some favourable outcomes regarding improvement in the oxidative status of blood and meat, indicating a positive effect on animal health. Therefore, the levels of anti-nutrients present should be managed to get the best results. *L*. *leucocephala* like *G. sepium* has a similar issue with anti-nutrients, with a special attention on mimosine that can also lower FI and potentially growth, even so this forage does have a noteworthy protein content and other minerals that can create a good product if unhindered by the effects of mimosine. Moreover, mimosine does have some positive effects on animal health, so the best solution would be to ensure pre-treatment that would keep the levels in check to derive the best outcome. Mulberry, in the same vein with *G. sepium* and *L*. *leucocephala* have been affected by anti-nutrients like tannins, cuamarins and saponins especially with higher inclusion levels that have depressed FI, along with cellulose and protein digestibility. Still, some of these anti-nutrients can provide positive health benefits, as seen with some of the other reviewed forages. The recommendation will also be to manage the levels of these bioactive compounds so that they don’t overshadow the potential of the nutrients contained in the forage. In the case of the widely studied Moringa, the positives have outweighed the negatives showing its dominance over the other alternatives, including an impressive nutritional content, highly digestible fibre and polyphenols that help to promote a robust product. Though this tree has demonstrated strong performance, this should be tempered with evidence of anti-nutrients that could limit, the animals’ progress. Overall, most of these forages have nutritional strengths that are mainly encumbered by anti-nutrients, which at the same time offer health benefits. Management of these phytochemicals would advance the incorporation of these sources in rabbit production.

## 9. Conclusions

In conclusion, the reviewed tropical fodder trees can be used as alternative sources in the diets of rabbits to bolster production. However, to effectively use these forages, special attention should be given to pre-treatment methods to minimize the effects of anti-nutritional metabolites contained in them.

## Data Availability

No new data were created or analyzed in this study. Data sharing is not applicable to this article.

## Abbreviations

GE: Gross Energy; DM: Dry Matter; OM: Organic Matter; CP: Crude Protein; EE: Ether Extract (Crude Fat); CF: Crude Fibre; NDF: Neutral Detergent Fiber; ADF: Acid Detergent Fiber; NFE: Nitrogen Free Extract; FI: Feed Intake; DFI: Daily Feed Intake; DWG: Daily Weight Gain; FBW: Final Body Weight; LBW: Live Body Weight; TFI: Total Feed Intake; TWG: Total Weight Gain; TBWG: Total Body Weight Gain; FCR: Feed Conversion Ratio; HDL: High Density Lipoprotein; LDL: Low Density Lipoprotein; CTTAD: Coefficients of Total Tract Apparent Digestibility of Diets; AST: Aspartate Amino Transferase; GPx: Glutathione Peroxidase; GSH: Glutathione; MDA: Malondialdehyde; TAC: Total Antioxidant Capacity; TBARS: Thiobarbituric Acid Reactive Substances; FA: Fatty Acids; NZW: New Zealand White; CW: California White; Chin: Chincilla; SFA: Saturated Fatty Acid; AFW: Average Final Weight; ADWG: Average Daily Weight Gain; g/d: grams/day
